# Michael William Blackburn Bradbury 1930–2013

**DOI:** 10.1186/2045-8118-10-26

**Published:** 2013-08-15

**Authors:** David J Begley, Hazel C Jones

**Affiliations:** 1 Institute of Pharmaceutical Science, School of Biomedical Sciences, King’s College London, Stamford Street, London, SE1 8WA, UK

## 

Mike Bradbury: Professor of Physiology, King’s College London 1977–1995, Emeritus 1995–2013.

Mike Bradbury was born on the 2nd of July 1930 in Capetown South Africa. He attended Sherborne School in Dorset and in 1949 was awarded an Open Scholarship to Christ Church, Oxford. He obtained an honours degree in Physiology in 1952 having been awarded *inter alia* a Theodore Williams Scholarship in Human Anatomy. Mike completed his medical training at St. Bartholomew’s Hospital in Smithfield, City of London. He subsequently graduated BM, BCh from the University of Oxford in 1956.

After graduating, he carried out research for the degree of DM in the laboratory of R.V. Coxon, Nuffield Department of Clinical Biochemistry and University Laboratory of Physiology, Oxford, and presented a thesis in 1962 entitled “Transfer and Distribution of Urea in the Body”. Part of this study was on the movement of urea into the brain [1]. This aspect of his early research was to dominate the rest of his scientific career and subsequent research into the regulation of the brain fluid environment and the transport of drugs, ions and other solutes across the brain microvasculature which forms the major component of the blood–brain barrier.

After Oxford, Mike returned to London to a post of Research Assistant, supported by the Medical Research Council, in the Department of Physiology at University College, where he worked with, and was highly influenced by, Hugh Davson [2]. Hugh was a pioneering figure in blood–brain barrier and cerebrospinal fluid research. Together they used the technique of ventriculo-cisternal perfusion adapted from the original method of Pappenheimer *et al.*[3] to quantify the transport of various substances into and out of the cerebrospinal fluid. This method is still in use today.

In 1965 he moved to Los Angeles, California, as Assistant Professor of Physiology University of California and Research Physiologist at Cedars-Sinai Medical Center, where he worked with Bill Oldendorf [4], a leader in the quantification of transport phenomena at the blood–brain barrier. Returning to the UK in 1968 he was appointed Senior Lecturer in Physiology, St Thomas’s Hospital Medical School, then Reader in Physiology, King’s College London in 1972, being made full Professor in 1977. During this period he made a continuous and vigourous contribution to Departmental and College life serving as Chairman of the Integration and Steering Committee developing a new undergraduate medical curriculum. His undergraduate lectures were always, lively, informative and entertaining. He was also active at the London University level serving both as Secretary and Chairman of the Board of Studies in Physiology, and also the Academic Advisory Boards of Science and Medicine. He became a member of the Physiological Society in 1964 and served on the Editorial Board of Journal of Physiology from 1981–1988.

Throughout his career, Mike published widely on the blood–brain barrier and transport phenomena across the cerebral microvasculature and also on the control of the brain extracellular fluid and cerebrospinal fluid composition. A major opus was the publication of his monograph volume “The Concept of the Blood–brain Barrier” in 1979 which reviewed and updated the entire field: a rapidly expanding area of research at this time [5]. This book was generally known as “the blue book” by its many adherents and readers on account of its striking blue dust jacket and binding. Its presence was a must on the bookshelves of colleagues and research students. At King’s, he established a thriving group and a laboratory which attracted a large number of scientists, colleagues and research students. During Mike’s career many visiting scientists spent some sabbatical time in his lab and these collaborations were invariably productive and usually led to publications and to lasting interactions. Amongst the many visitors and collaborators were Barbora Štulková [6], Norman Saunders [7]. Hans Bronsted [8], Jill Cremer [9], Joe Fenstermacher [10], Mike Carey [11], Helen Cserr [12], Gabe Pinter [13], Sue Lightmann [13], and Gary Rosenberg [14].

Mike developed a number of techniques to quantify transport at the blood–brain barrier. He wanted to determine accurately the transport of ions into the brain using a method to maintain constant blood levels by continuous intravenous infusion: a technically difficult task. During a visit by colleagues Cliff Patlak and Ron Blasberg, from the National Institutes of Health, Bethesda, Maryland, a lively discussion developed on whether it was possible to mathematically compensate for a decreasing concentration of solute in blood. The next week when Cliff and Ron were visiting the lab of Christian Crone in Copenhagen, Cliff presented the mathematical solution to the problem on a blackboard. In the audience in Copenhagen was Albert Gjedde who was the first person to apply the analytical technique to Positron Emission Tomography studies in animals and man [15]. Mike had provided the intellectual concept and stimulus and Cliff the mathematical skills. Thus the method of multi-time-point regression analysis was born. This approach still remains the basis of the most accurate and sensitive methods for measuring solute uptake by the brain and is acknowledged as being the “Gold Standard” for comparative studies of brain solute uptake. It is also still called the Patlak plot [16].

When Mike retired, a Festschrift was held in his honour at King’s attended by over one hundred of his colleagues and former students, a mark of his significant international reputation. The collected conference proceedings were published in a volume entitled “New Concepts of a Blood–brain Barrier” [17] in honour of his earlier volume and his numerous contributions to the field. Mike had a sharp and very enquiring mind which was very open to radical ideas. At one point, in spite of some personal scepticism, he was advising the Physics Department on whether it was possible for practitioners like Uri Geller to generate electromagnetic potentials which made his apparent feats possible and whether these could be measured, and was also investigating the possible mechanisms of acupuncture.

In retirement, Mike used his Professor Emeritus status to the full: he was a regular visitor to the labs of colleagues for lively discussion and he was an invaluable mentor and critic to a host of research students. Mike’s characteristic and penetrating laugh always announced his presence. A retirement project was writing a volume on the colonization of North America. Sadly this project was not finished. Privately, Mike was a wonderful host to his friends and colleagues and also an enthusiastic yachtsman. His wife Anne survives him together with a daughter Joanna and two sons Nicholas and Timothy. Mike died peacefully on the 9th of February 2013 in Blandford, Dorset. Mike is remembered fondly by his numerous colleagues and collaborators and by generations of Medical and Science students.

**Figure 1:**
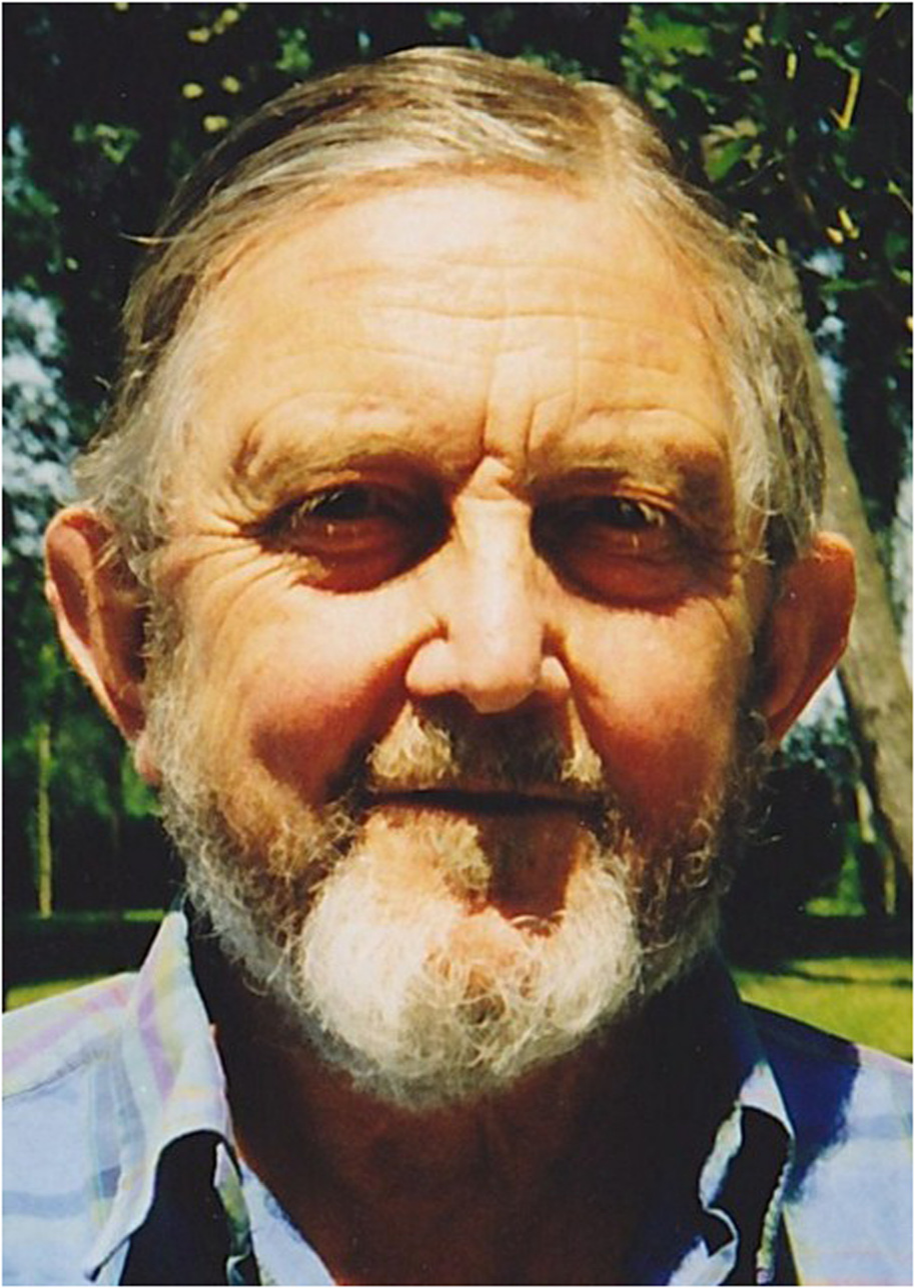
Michael William Blackburn Bradbury.
